# Genetic polymorphisms and haplotypes of ERCC1 and ERCC2 associated with quality of life, depression, and anxiety status among patients with lung cancer

**DOI:** 10.1186/s12885-021-08570-5

**Published:** 2021-07-21

**Authors:** Yunxiang Tang, Ruike Zhang, Yinan Li, Shuyu Xu, Hao Wang, Jingzhou Xu, Lei Xiao, Yajing Wang, Jing Du, Yujia Huang, Tong Su

**Affiliations:** Department of Medical Psychology, College of Psychology, Naval Medical University, 800 Xiangyin Rd., Shanghai, 200433 China

**Keywords:** ERCC1, ERCC2, Single nucleotide polymorphism, Quality of life, Depression, Anxiety, Lung cancer

## Abstract

**Background:**

Patients with lung cancer (LC) have a poor quality of life (QoL) and easily suffer from psychological diseases. Previous studies focused less on the relationship between genetic factors and QoL, depression, and anxiety status in LC patients. The current study is intended to explore the relationship between SNPs and haplotypes of ERCC1 and ERCC2 and the QoL, depression and anxiety status of patients with LC.

**Methods:**

QoL, depression and anxiety status were assessed in 291 LC patients using the European Organization for Research and Treatment of Cancer (EORTC) Core Quality of Life Questionnaire (QLQ-C30), EORTC Quality of Life Questionnaire-Lung Cancer 13 (QLQ-LC13), SDS and SAS. Nine tag SNPs of ERCC1 and ERCC2 were detected using an improved multiplex ligation detection reaction (iMLDR) technique. Haplotype analysis was conducted using the software Haploview 4.2. The association between SNPs or haplotypes and QoL or depression or anxiety in LC patients was analyzed by regression analysis.

**Results:**

ERCC1 rs11615 was associated with emotional functioning (*P* = 0.027), and ERCC1 rs3212986 was associated with anxiety scores (*P* = 0.018). ERCC1 rs762562-rs3212986 haplotype was associated with cognitive function (*P* = 0.029), somatic function (*P* = 0.014) and dysphagia (OR = 3.32, *P* = 0.044). Patients with ERCC1 rs3212986-rs11615 AG haplotype had worse cognitive function (adjusted Beta = − 5.42) and somatic function (adjusted Beta = − 6.55) and had severer symptoms of loss of appetite (adjusted OR = 1.67) and dysphagia (adjusted OR = 4.43) (All adjusted *P* < 0.05). ERCC2 rs13181-rs3916874-rs238416 haplotype was associated with emotional functioning (*P* = 0.035), pain at other sites (OR 1.88, *P* = 0.014), chest pain (OR 0.42, *P* = 0.02), dysphagia (OR 2.82, *P* = 0.048), and anxiety status (OR 0.23, *P* = 0.009).

**Conclusion:**

After adjustment for environmental factors, SNPs and haplotypes of ERCC1 and ERCC2 were associated with different domains of QoL, depression and anxiety in LC patients.

**Supplementary Information:**

The online version contains supplementary material available at 10.1186/s12885-021-08570-5.

## Background

Lung cancer, as one of the malignant neoplasms, has the highest incidence and mortality rate worldwide. There were 2.1 million lung cancer cases identified in 2018 in the world, accounting for 11.6% of all cancer cases. 1.8 million lung cancer-related deaths accounted for 18.4% of all cancer-related deaths [1]. The incidence rate of lung cancer is increasing year by year, and it has become one of the burdens of the major disease in China [2]. Most patients are in their middle or advanced stage at the first diagnosis of lung cancer with a poor prognosis and a low five-year survival rate [3]. Chemotherapy is the main treatment for advanced lung cancer, which is characterized by long treatment cycles and serious adverse reactions. It also causes heavy financial burdens. The physical and mental health and even families of patients are negatively affected. Compared with other tumors, lung cancer patients have a poorer quality of life (QoL) [4]. Therefore, for lung cancer patients, the goal of treatment is not only to improve the survival rate and survival time but also to improve the quality of life of patients with limited survival periods.

QoL refers to the self-evaluated physiological, psychological, and social feelings about the disease and its treatment by patients. The assessment of QoL has important value for clinical research of lung cancer, which can be used to evaluate the therapeutic effect, screen chemotherapy drugs, analyze the prognosis and long-term survival status. According to the systematic review published in Lancet Oncol in 2019, among 44 studies published between 2006 and 2018, the methods used for prognostic factor analysis are more standardized and rigorous than before. Forty-one (93%) trials reported at least one area of QoL as an independent prognostic factor. The most common prognostic factors were physiological function (17 studies, 39%) and total health status (15 studies, 34%). These findings highlight the value of QoL as an independent prognostic factor in cancer research [5]. The basis of improving the QoL is to clarify the influencing factors of QoL with lung cancer. At present, the influencing factors of QoL with lung cancer have not been fully defined.

The QoL of lung cancer is affected by many factors. In the past, most studies focused on demographic, sociological factors, such as gender, age, marriage, and factors of clinical characteristics and treatment. However, the research results of different diseases and different regions are not completely consistent. This may be due to differences in the study population and disease characteristics or other factors (such as genetic factors) that affect the QoL. With the intersection and integration of molecular biology, psychology, and epidemiology, most researchers agree that individual physiology, psychology, and behavior are the result of both genetic and environmental factors. In 2004, Hampton et al. proposed that the QoL has a certain genetic basis, and gene technology should be integrated into the study of QoL [6, 7]. In 2014, a review summarized the biological pathways, candidate genes, and molecular markers related to the QoL. The results showed that different areas of QoL were related to different pathways. Fatigue was related to inflammatory pathways. The pain was related to inflammation and neural transmission pathways. Depression was related to neurotransmitters and neural plasticity. Oxytocin-related genes and genes related to serotonin and dopamine pathways play a role in social functions [8].

Although more than 50 candidate genes of multiple pathways have been found to be related to pain, fatigue, emotional symptoms, and other areas of QoL, the predictors of QoL in lung cancer have not been fully understood. The function of ERCC1 and ERCC2 genes are closely related to the nucleotide excision repair (NER) pathway of the DNA repair system. There are few studies on the relationship between single nucleotide polymorphisms (SNPs) of ERCC1 and ERCC2 and QoL in lung cancer patients. ERCC1 can form a dimer with xeroderma protein F (XPF), recognize and remove the mismatch region in the DNA chain, repair and connect the nucleotide fragments after excision, restore the normal DNA structure, which plays an important role in maintaining the stability and integrity of DNA in vivo [9]. ERCC2 is a DNA helicase, which is an integral component of TFIIH, playing an essential role in transcription and NER. The loss of ERCC2 function will lead to the failure of the transcription factor complex to identify DNA damage site and excising and repairing bases accurately, resulting in the accumulation of DNA damage. SNPs of ERCC1 and ERCC2 genes may affect the protein expression level and activity and affect the resection and repairability of the NER system, which are related to lung cancer susceptibility, chemotherapy effect and toxicity of platinum drugs, and prognosis of lung cancer [10–14]. Therefore, ERCC1 and ERCC2 SNPs may be associated with the QoL of lung cancer patients.

The current study is intended to examine the effect of SNPs and haplotypes of ERCC1 and ERCC2 on the QoL, depression and anxiety of LC patients with demographic and clinical characteristics correction. We hope it will provide clues for early identification of patients with poor QoL, depression or anxiety.

## Methods

### Subjects

The subjects of the study were patients with primary lung cancer admitted to the respiratory department of Changhai Hospital Affiliated with Naval Medical University between November 2016 and October 2018. The inclusion criteria were: patients (1) were diagnosed with primary lung cancer; (2) could complete the surveys themselves or with help; (3) were aware of their illness conditions. The exclusion criteria were: patients (1) had the pathological type of small cell lung cancer; (2) had been diagnosed with other cancers; (3) with severe psychiatric or somatic illnesses.

This study was approved by the ethics committee of Naval Medical University. Subjects were informed in detail about the experiment and gave their consent. The data of patients used in the current study have been partly used in a previous study addressing associations between QoL and survival of lung cancer patients and the BRCA1 gene [15].

### Genotyping

All SNPs information of ERCC1 and ERCC2 were downloaded from the HapMap database using Haploview4.2 software. The conditions were set as CHB, R^2^ > 0.8, MAF > 0.10. Three tag SNPs of ERCC1 (rs11615, rs762562, rs3212986) and six tag SNPs of ERCC2 (rs13181, rs171140, rs3916874, rs50872, rs50871, rs238416) were obtained after calculation.

Wizard Genomic DNA Purification Kit (Promega, Madison, Wisconsin, USA) was used to extract genomic DNA. The improved multiplex ligation detection reaction (iMLDR) technique was used to perform SNP genotyping [16].

### Assessment of quality of life, anxiety and depression status

During hospitalization, the demographic, sociological, clinical characteristics and treatment of patients were collected by consulting medical records or interviewing patients. After obtaining the consent of patients, the European Organization for Research and Treatment of Cancer (EORTC) Core Quality of Life Questionnaire (QLQ-C30) and EORTC Quality of Life Questionnaire-Lung Cancer 13 (QLQ-LC13) were used to evaluate the quality of life of lung cancer patients. At the same time, the self-rating Anxiety Scale (SAS) [17] and self-rating Depression Scale (SDS) [18] were used to assess the psychological status of patients further.

Both QLQ-C30 and QLQ-LC13 are self-rated questionnaires. The QLQ-C30 comprises 15 scales, including five functional scales (physical, role, cognitive, emotional, and social), three symptom scales (fatigue, pain, and nausea and vomiting), a global health and quality-of-life scale, as well as six single items (dyspnea, appetite loss, sleep disturbance, constipation, and diarrhea), with a total of 30 items [19]. The QLQ-LC13 is a modular supplement to the QLQ-C30, consisting of 13 items assessing cancer-related symptoms and side effects from cancer treatment. It contains ten symptoms, including shortness of breath, cough, hemoptysis, oral ulcer, dysphagia, peripheral neuropathy, alopecia, chest pain, arm/shoulder pain and pain in other parts [20]. Raw scores of each domain were standardized using linear transformation for comparison. The higher the functional scales and the global health scale, the better the functional status and quality of life. On the contrary, the higher the scores of symptom scales and single items, the more obvious the symptoms and the worse the quality of life.

Both SAS and SDS consist of 20 items scored on a Likert scale of 1 to 4. The total scores were standardized. SAS standard score ≥ 50 is considered to have anxiety symptoms, with 50–60 rated as mild anxiety, 61–70 as moderate anxiety, and > 70 as severe anxiety [17]. Standard scores < 53 indicate no depression for the SDS, 53–62 mild, 63–72 moderate, and > 72 severe depression [18].

### Statistical analysis

Descriptive statistics of patients’ general condition, QoL, depression, anxiety, and genotypes were calculated. The scores for the symptom scales and single items of the QoL questionnaire (QLQ-C30 and QLQ-LC13) were skew distribution. According to the scale instructions, the scales were dichotomized as mild symptoms (< 50) and severe symptoms (≥50), and the frequency of symptoms was calculated [21].

Linkage disequilibrium analysis of all tag SNPs in the genes was performed with Haploview v4.2 [22] to obtain statistically associated SNPs -- haplotypes. The main model of association analysis is the additive model, which takes major alleles or major haplotypes as reference. In addition to the additive model, the dominant model was used in exploring associations between a single SNP and Quality of Life. Linear regression analysis was used to analyze SNP or haplotype and continuous variables (scores of functional scales, global health scale, SAS and SDS). Logistic regression analysis was used for association analysis between SNP or haplotype and dichotomized variables (symptoms, single items and status of depression and anxiety). The environmental factors were included in regression models as covariates for adjustment, including sex, age, occupation, marriage, education, number of children, smoking history, drinking history, medical insurance, pathological types, clinical stages, metastasis, concurrent symptoms, operation history, and chemotherapy. Statistical significance was corrected with Bonferroni methods. Statistical analyses were performed with SPSS 25.0 (Chicago, IL, USA). All statistical tests were performed using a two-sided probability test, and differences were considered statistically significant at *P* < 0.05.

## Results

### Patient characteristics

The final number of lung cancer patients included in the statistical analysis was 291. The age ranged from 24 to 89 years, with a mean age of 60.02 ± 10.94 years and 59.1% of those ≥60 years. There were 203 males (69.8%). The vast majority were married (94.8%), most had only one child (62.9%), and most had a history of smoking (74.9%) and alcohol consumption (74.2%). The subjects were all patients with non-small cell lung cancer, with the main types of pathology being adenocarcinoma (47.8%) and squamous carcinoma (21.6%), and the main clinical stages being stage IV (66.7%) and stage III (24.1%). Most patients (98.3%) received chemotherapy without surgery. 187 (64.3%) patients had less than four sessions of chemotherapy, and 104 (35.7%) patients had four or more sessions of chemotherapy. Patients received mainly platinum-based chemotherapy regimens in cycles of 3 to 4 weeks. These patients have not been treated with targeted therapies or immunotherapy.

Patient information was described in detail in a previous study [15].

### SNPs and haplotypes

The distribution of tagSNPs genotypes of ERCC1 and ERCC2 are shown in (Table 1). Three SNP combinations of ERCC1 and ERCC2 were obtained by linkage disequilibrium analysis: ERCC1 rs762562-rs3212986, ERCC1 rs3212986-rs11615, ERCC2 rs13181-rs3916874-rs238416 (Table 2). The haplotypes with the largest number were used as a reference for subsequent analysis.

**Table 1:** Genotype distribution of tagSNPs in ERCC1 and ERCC2

Gene	SNPs	n	%
ERCC1	rs11615		
	GG (CC)	159	54.6
	GA (CT)	115	39.5
	AA (TT)	17	5.8
ERCC1	rs762562		
	AA	95	32.6
	GA	148	50.9
	GG	48	16.5
ERCC1	rs3212986		
	CC	125	43.0
	CA	140	48.1
	AA	26	8.9
ERCC2	rs13181		
	TT	245	84.2
	GT	43	14.8
	GG	3	1.0
ERCC2	rs171140		
	AA	86	29.6
	CA	140	48.1
	CC	65	22.3
ERCC2	rs238416		
	CC	82	28.2
	CT	152	52.2
	TT	57	19.6
ERCC2	rs3916874		
	CC	192	66.0
	GC	87	29.9
	GG	12	4.1
ERCC2	rs50871		
	AA	143	49.1
	CA	120	41.2
	CC	28	9.6
ERCC2	rs50872		
	GG	194	66.7
	GA	88	30.2
	AA	9	3.1

**Table 2:** Haplotypes of ERCC1, ERCC2 gene

Gene	SNPs	Haplotype	Quantity	Frequency*
ERCC1	rs762562-rs3212986	GC (ref)	243	0.4175
ERCC1	rs762562-rs3212986	AA	191	0.3282
ERCC1	rs762562-rs3212986	AC	147	0.2526
ERCC1	rs3212986-rs11615	CG (ref)	242	0.4158
ERCC1	rs3212986-rs11615	AG	191	0.3282
ERCC1	rs3212986-rs11615	CA	148	0.2543
ERCC2	rs13181-rs3916874-rs238416	TCT (ref)	266	0.4570
ERCC2	rs13181-rs3916874-rs238416	TCC	156	0.2680
ERCC2	rs13181-rs3916874-rs238416	TGC	111	0.1907
ERCC2	rs13181-rs3916874-rs238416	GCC	49	0.0842

Note: when calculating the frequency, the numerator = the corresponding number of haplotype, denominator = 2 * sample size. * means multiplication

### Quality of life and depression and anxiety status

The standardized scores of the 291 study participants for each domain of the quality of life scale were mostly close to the reference value “EORTC QLQ-C30 Reference Values” [21], but the score for the economic hardship domain (mean = 53.38, SD = 36.92) was significantly higher than the reference values (mean = 17.4, SD = 28.9). The scores of the study subjects were described in detail in the previous study [15].

Because of the skewed distribution of symptom scores, a cut-off of 50 was converted to a dichotomous variable (symptom light and symptom heavy) for subsequent analysis. The number and proportion of symptom heaviness were: C30 fatigue symptom heaviness 79 (27.1%), C30 nausea and vomiting symptom heaviness 59 (20.3%), C30 pain symptom heaviness 84 (28.9%), C30 shortness of breath symptom heaviness 73 (25.1%), C30 insomnia symptom heaviness 87 (29.9%), C30 loss of appetite symptom heaviness 82 (28.2%), C30 constipation in 59 (20.3%), C30 diarrhea in 17 (5.8%), C30 economic difficulties in 144 (49.5%), LC13 cough in 71 (24.4%), LC13 hemoptysis in 19 (6.5%), LC13 shortness of breath in 38 (13.1%), LC13 mouth ulcer in 18 (6.2%), LC13 dysphagia 14 (4.8%), LC13 peripheral neuropathy 24 (8.2%), LC13 alopecia 45 (15.5%), LC13 chest pain 50 (17.2%), LC13 arm/shoulder pain 50 (17.2%), LC13 pain at other sites 49 (16.8%).

The mean score (standardized score, mean ± SD) on the SDS scale was 52.28 ± 11.69, and the mean score (standardized score, mean ± SD) on the SAS scale was 43.49 ± 9.13 in 291 study subjects. 142 (48.8%) patients had a depressive state, and 67 (23.0%) patients had an anxious state, of whom most were mild depression (102/142), and mild anxiety (58/67), and depression and anxiety were significantly correlated (*p* < 0.001), 38.0% (54/142) of the depressed patients had a comorbid anxiety state and 80.6% (54/67) of the anxious patients had a comorbid depression state.

### The association of SNPs to the quality of life, depression and anxiety

Both ERCC1 and ERCC2 have SNP loci associated with quality of life, anxiety and depression in lung cancer, mainly focusing on ERCC1rs11615 and rs3212986, associated with multiple domains of quality of life (Table 3).

**Table 3:** Associations between single SNP of ERCC1 and ERCC2 gene and Quality of Life, depression and anxiety (unadjusted and adjusted for Environmental factors)

Area	SNP	Gene	Ref/Alt	Model	Beta (95%CI) ^a^or OR (95%CI)^b^	*P*	Bofferoni adjusted	Environmental factors correction		
								Beta (95%CI) ^a^or OR (95%CI)^b^	*P*	Bofferoni adjusted
C30 Emotional functions	rs11615	ERCC1	G/A	Additive	6.34 (2.09–10.59)	**0.004**	**0.036**	6.85 (2.38–11.31)	**0.003**	**0.027**
C30 Fatigue	rs11615	ERCC1	G/A	Additive	0.62 (0.39–0.98)	**0.041**	0.369	–	–	–
LC13 Dysphagia	rs11615	ERCC1	G/A	Additive	0.20 (0.047–0.88)	**0.033**	0.297	0.18 (0.04–0.87)	**0.033**	0.297
SAS Anxiety standard score	rs11615	ERCC1	G/A	Additive	−1.94 (−3.66--0.22)	**0.028**	0.252	−2.06 (−3.85--0.27)	**0.025**	0.225
C30 Cognitive functions	rs3212986	ERCC1	C/A	Additive	−5.72 (−10.15--1.31)	**0.012**	0.108	−6.18 (−10.74--1.62)	**0.008**	0.072
C30 Physical functions	rs3212986	ERCC1	C/A	Additive	−5.43 (−9.68--1.18)	**0.013**	0.117	−5.98 (−10.40--1.56)	**0.008**	0.072
C30 Fatigue	rs3212986	ERCC1	C/A	Additive	1.52 (1.02–2.28)	**0.042**	0.378	–	–	–
LC13 Dysphagia	rs3212986	ERCC1	C/A	Additive	2.31 (1.02–5.22)	**0.045**	0.405	4.87 (1.43–16.62)	**0.011**	0.099
SDS depression standard score	rs3212986	ERCC1	C/A	Dominant	3.07 (0.37–5.77)	**0.026**	0.234	2.96 (0.21–5.71)	**0.036**	0.324
SAS Anxiety standard score	rs3212986	ERCC1	C/A	Dominant	3.09 (1.00–5.18)	**0.004**	**0.036**	3.41 (1.23–5.57)	**0.002**	**0.018**
SAS Anxiety State	rs3212986	ERCC1	C/A	Dominant	1.90 (1.06–3.39)	**0.030**	0.270	2.03 (1.11–3.73)	**0.022**	0.198
LC13 Pain in other parts	rs762562	ERCC1	A/G	Additive	1.60 (1.02–2.51)	**0.042**	0.378	1.73 (1.04–2.86)	**0.034**	0.306
LC13 Chest pain	rs3916874	ERCC2	C/G	Additive	0.48 (0.25–0.93)	**0.029**	0.261	–	–	–
SAS Anxiety State	rs13181	ERCC2	T/G	Dominant	0.28 (0.095–0.80)	**0.018**	0.162	0.22 (0.07–0.66)	**0.007**	0.063

Ref/Alt:Refer to allele (wild-type allele) / mutant allele. Model:Additive model or Dominant model. Covariates refer to environmental factors including:sex, age, occupation, marriage, education, number of children, smoking history, drinking history, medical insurance, pathological types, clinical stages, metastasis, concurrent symptoms, operation history, and chemotherapy. ^a^ β and 95% confidence interval (*CI*) were reported (Linear regression was used for general health condition and functioning domains, anxiety and depression). ^b^ odds ratio (*OR*) and 95% CI were reported (Logistic regression was used for symptoms domains, anxiety and depression)

However, after adjustment for environmental factors and multiple tests (Bonferroni adjusted), only ERCC1 rs11615 was significantly associated with emotional function (environment adjusted beta = 6.85, 95% CI = 2.38–11.31, Bonferroni adjusted *P* = 0.027), and ERCC1 rs3212986 was significantly correlated with anxiety score (environment adjusted beta = 3.41, 95% CI = 1.23–5.57, Bonferroni adjusted *P* = 0.018). In other words, patients with ERCC1 rs11615 A allele had a better emotional function, while patients with ERCC1 rs3212986 A allele had severer anxiety than those without the allele.

### The Association of Haplotypes to the quality of life, depression and anxiety

Both ERCC1 and ERCC2 haplotypes are associated with multiple domains of quality of life in lung cancer patients (Table 4).

**Table 4:** Associations between ERCC1 and ERCC2 gene haplotypes and Quality of Life, depression and anxiety (unadjusted and adjusted for Environmental factors)

Area	Gene	SNPs	Haplotype	*P*	Beta (95%CI)^a^ or OR (95%CI)^b^	Environmental factors correction	
						*P*	Beta (95%CI)^a^ or OR (95%CI)^b^
C30 Cognitive functions	ERCC1	rs762562-rs3212986	AA	**0.038**	−4.87 (− 9.45--0.29)	**0.029**	− 5.34 (− 10.13--0.56)
			AC	0.058	4.66 (−0.14–9.47)	0.083	4.47 (− 0.56–9.50)
			GC (ref)	–	–	–	–
C30 Physical functions	ERCC1	rs762562-rs3212986	AA	**0.019**	−5.33 (−9.75--0.90)	**0.014**	− 5.89 (−10.53--1.25)
			AC	0.239	2.77 (−1.83–7.38)	0.202	3.22 (−1.71–8.14)
			GC (ref)	–	–	–	–
LC13 Dysphagia	ERCC1	rs762562-rs3212986	AA	0.165	1.80 (0.79–4.10)	**0.044**	3.32 (1.03–10.66)
			AC	0.063	0.31 (0.09–1.06)	0.133	0.33 (0.08–1.40)
			GC (ref)	–	–	–	–
C30 Emotional functions	ERCC1	rs3212986-rs11615	AG	0.490	−1.52 (−5.83–2.79)	0.490	−1.59 (−6.12–2.93)
			CA	**0.007**	6.04 (1.72–10.36)	**0.005**	6.61 (2.08–11.15)
			CG (ref)	–	–	–	–
C30 Cognitive functions	ERCC1	rs3212986-rs11615	AG	**0.027**	−5.27 (−9.90--0.63)	**0.028**	−5.42 (− 10.24--0.60)
			CA	0.075	4.38 (−0.43–9.19)	0.060	4.77 (−0.18–9.72)
			CG (ref)	–	–	–	–
C30 Physical functions	ERCC1	rs3212986-rs11615	AG	**0.009**	−6.06 (−10.55--1.57)	**0.007**	−6.55 (− 11.24--1.86)
			CA	0.178	3.17 (−1.43–7.77)	0.125	3.80 (−1.04–8.65)
			CG (ref)	–	–	–	–
C30 Appetite loss	ERCC1	rs3212986-rs11615	AG	**0.037**	1.57 (1.03–2.40)	**0.025**	1.67 (1.07–2.61)
			CA	0.708	1.09 (0.70–1.68)	0.956	0.99 (0.62–1.57)
			CG (ref)	–	–	–	–
LC13 Dysphagia	ERCC1	rs3212986-rs11615	AG	0.068	2.18 (0.94–5.05)	**0.019**	4.43 (1.28–15.39)
			CA	**0.027**	0.19 (0.04–0.82)	**0.035**	0.17 (0.032–0.88)
			CG (ref)	–	–	–	–
SAS Anxiety standard score	ERCC1	rs3212986-rs11615	AG	0.130	1.34 (−0.39–3.07)	0.076	1.63 (−0.16–3.41)
			CA	**0.014**	−2.25 (−4.02- -0.47)	**0.023**	−2.18 (−4.05- -0.32)
			CG (ref)	–	–	–	–
C30 Emotional functions	ERCC2	rs13181-rs3916874-rs238416	GCC	**0.047**	6.81 (0.125–13.50)	**0.035**	7.60 (0.60–14.61)
			TCC	0.411	−1.75 (−5.91–2.42)	0.248	−2.60 (−7.00–1.80)
			TGC	0.199	3.17 (− 1.66–8.01)	0.124	4.00 (− 1.08–9.09)
			TCT (ref)	–	–	–	–
LC13 Chest pain	ERCC2	rs13181-rs3916874-rs238416	GCC	0.899	0.95 (0.45–2.03)	0.712	1.18 (0.50–2.77)
			TCC	0.705	1.09 (0.69–1.74)	0.736	1.10 (0.64–1.86)
			TGC	**0.018**	0.44 (0.22–0.87)	**0.020**	0.42 (0.21–0.87)
			TCT (ref)	–	–	–	–
LC13 Pain in other parts	ERCC2	rs13181-rs3916874-rs238416	GCC	0.367	0.66 (0.27–1.61)	0.198	0.52 (0.19–1.41)
			TCC	**0.042**	1.59 (1.02–2.50)	**0.014**	1.88 (1.14–3.12)
			TGC	0.093	0.57 (0.29–1.10)	0.145	0.58 (0.28–1.21)
			TCT (ref)	–	–	–	–
LC13 Dysphagia	ERCC2	rs13181-rs3916874-rs238416	GCC	0.491	0.49 (0.06–3.73)	0.546	0.44 (0.03–6.32)
			TCC	0.062	2.10 (0.96–4.55)	**0.048**	2.82 (1.01–7.85)
			TGC	0.758	1.18 (0.42–3.33)	0.451	0.60 (0.16–2.28)
			TCT (ref)	–	–	–	–
SAS Anxiety state	ERCC2	rs13181-rs3916874-rs238416	GCC	**0.018**	0.28 (0.10–0.80)	**0.009**	0.23 (0.08–0.69)
			TCC	0.401	1.20 (0.79–1.81)	0.479	1.18 (0.75–1.85)
			TGC	0.188	0.70 (0.41–1.19)	0.211	0.69 (0.39–1.23)
			TCT (ref)	–	–	–	–

Covariates refer to environmental factors including:sex, age, occupation, marriage, education, number of children, smoking history, drinking history, medical insurance, pathological types, clinical stages, metastasis, concurrent symptoms, operation history, and chemotherapy

When calculating the frequency, the molecule = the corresponding haplotype number, the denominator of the reference haplotype = 2 * (the sample number that contains the reference haplotype), the denominator of none reference haplotype = 2 * (the sample number that contains the reference and the current haplotypes). * means multiplication

^a^β and 95% confidence interval (*CI*) were reported (Linear regression was used for general health condition and functioning domains, anxiety and depression). ^b^odds ratio (*OR*) and 95% CI were reported (Logistic regression was used for symptoms domains, anxiety and depression)

ERCC1 rs762562-rs3212986 haplotype is associated with cognitive function, somatic function and dysphagia. Compared with GC haplotype, ERCC1 rs762562-rs3212986 AA haplotype was significantly associated with worse cognitive function (adjusted beta = − 5.34, 95% CI = − 10.13-0.56, adjusted *P* = 0.029), somatic function (adjusted beta = − 5.89, 95% CI = − 5.89, 95% CI = − 10.53-1.25, adjusted *P* = 0.014), and severer in dysphagia symptoms (adjusted OR = 3.32, 95% CI = 1.03–10.66, adjusted *P* = 0.044) after correction for environmental factors, indicating that ERCC1 rs762562-rs3212986 AA haplotype is a risk factor for quality of life in lung cancer patients.

ERCC1 rs3212986-rs11615 haplotype was associated with emotional function, cognitive function, somatic function, loss of appetite, dysphagia and anxiety scores. After adjustment for environmental factors, compared with CG haplotype, patients with ERCC1 rs3212986-rs11615 AG haplotype had worse cognitive function (adjusted Beta = − 5.42, adjusted *P* = 0.028) and somatic function (adjusted Beta = − 6.55, adjusted *P* = 0.007), and had severer symptoms of loss of appetite (adjusted OR = 1.67, adjusted *P* = 0.025) and dysphagia (adjusted OR = 4.43, adjusted *P* = 0.019), which indicated that ERCC1 rs3212986-rs11615 AG haplotype is a risk factor of quality of life in patients with lung cancer. On the other hand, compared with CG haplotype, ERCC1 rs3212986-rs11615 CA haplotype were associated with better emotional functions (adjusted Beta = 6.61, adjusted *P* = 0.005), mild dysphagia (adjusted OR = 0.035, adjusted *P* = 0.017), and lower anxiety score (adjusted Beta = − 2.18, adjusted *P* = 0.023) after environmental factors corrections, suggesting that ERCC1 CA haplotype is a protective factor of quality of life and anxiety in patients with lung cancer.

ERCC2 rs13181-rs3916874-rs238416 haplotype was associated with emotional function, pain in other parts, chest pain, dysphagia and anxiety. After adjusting for environmental factors, GCC haplotype compared with the TCT haplotype was correlated with better emotional function (adjusted beta = 7.60, adjusted *P* = 0.035) and lower anxiety risk (adjusted OR = 0.23, adjusted *P* = 0.009). In addition, chest pain was mild in patients with a copy of the TGC haplotype (adjusted OR = 0.42, adjusted *P* = 0.02). In contrast, severe pain in other parts (adjusted OR = 1.88, adjusted *P* = 0.014) and dysphagia (adjusted OR = 2.82, adjusted *P* = 0.048) were more severe in lung cancer patients with TGC haplotype.

## Discussion

According to the 2015 National Comprehensive Cancer Network (NCCN) guidelines for the treatment of non-small cell lung cancer, platinum-based chemotherapy is the first-line treatment for patients with advanced lung cancer [23]. Platinum drugs play an anti-tumor role mainly through the introduction of intra-chain and inter-chain cross-linking to destroy tumor cell DNA, leading to cell death [24]. Resistance to platinum-based drugs often leads to a poor prognosis. DNA repair pathway is the key molecular mechanism of resistance to platinum-based drugs. ERCC1 and ERCC2 play an indispensable role in the NER pathway of DNA repair and are related to clinical therapeutic effects and prognosis [12, 14].

ERCC1 activity was affected by ERCC1 gene polymorphism. Among the polymorphisms related to the absorption, metabolism, cytotoxicity, and excretion of platinum drugs, ERCC1 gene C118T (rs11615) and C8092A (rs3212986) is the most predictive characteristic SNP. A systematic review on gene variation and cisplatin toxicity showed that ERCC1 rs11615, rs3212986, and ERCC2 rs13181 were associated with cisplatin nephrotoxicity [25]. However, due to the heterogeneity of the researchers and the difference in chemotherapy regimens, the results of many studies on the predictive ability of ERCC1 polymorphism are not consistent [24]. At present, there are few studies on the effect of ERCC1 gene polymorphism on the quality of life of lung cancer patients.

The results showed that ERCC1 rs11615 was significantly correlated with emotional function (environment adjusted beta = 6.85, Bonferroni adjusted *P* = 0.027), and ERCC1 rs3212986 was significantly correlated with anxiety score (environment adjusted beta = 3.41, Bonferroni adjusted *P* = 0.018). In other words, patients with ERCC1 rs11615 A allele had better emotional functions, while patients with ERCC1 rs3212986 A allele had severer anxiety than those with the allele. Studies have shown that patients with ERCC1 rs11615 AA have better chemotherapy effects and longer progression-free survival, while patients with A allele of ERCC1 rs3212986 have a poor response to chemotherapy and shorter progression-free survival [26, 27]. It may be because the lung cancer patients with ERCC1 rs11615 A allele have better chemotherapy effects and longer survival time, which is conducive to maintain a good emotional state, so it reflects better emotional function and quality of life. However, the patients with ERCC1 rs3212986 A allele could not effectively relieve the symptoms due to poor chemotherapy effect, and their cognitive and physical functions were affected, which led to a higher anxiety level.

Previous studies on tumor susceptibility found that although single SNPs were not significantly associated with tumor risk, haplotypes containing one or more functional SNPs have significant associations with tumor susceptibility [13]. For example, two haplotypes containing the ERCC2 rs3916874 G allele were closely related to the risk of lung cancer, rs13181-rs3916874-rs238415 AGG haplotypes are associated with an increased risk of pancreatic cancer [28]. Studies on SNP and chemotherapy adverse reactions also found that the combination of genetic markers may be a better at-risk prediction [29]. In this study, although the correlation between a single ERCC2 SNP and quality of life was not significant after multiple test correction, the haplotype rs13181-rs3916874-rs238416 composed of three tagSNPs of ERCC2 gene was significantly associated with some areas of quality of life in patients with lung cancer. GCC haplotype was found to be correlated with better emotional function and lower anxiety risk. Lighter chest pain symptoms were found in patients with the TGC haplotype, while pain in other parts and dysphagia were severe in patients with TCC haplotype. ERCC1 rs3212986-rs11615 AG haplotype was associated with poor cognitive function and somatic function, severe symptoms of loss of appetite, and dysphagia, while LC patients having a copy of CA haplotype had better emotional functions, mild dysphagia, and lower anxiety score. ERCC1 rs762562-rs3212986 AA haplotype was found to be significantly correlated with poor cognitive and somatic functions and severe dysphagia. These results may reflect the correlation of polymorphisms and haplotypes of different genes with various domains of quality of life and suggest their significance in predicting the quality of life and prognosis of patients.

Neuropsychologic abnormalities such as anxiety and depression are common in cancer patients [30]. The study results showed that 48.8% of lung cancer patients had depression, 23.0% of patients had anxiety, most were mild depression (102/142) and mild anxiety (58/67). And depression and anxiety were significantly correlated. There is increasing evidence that genetic polymorphisms may lead to different susceptibility to psychoneurosis [31]. Catechol-O-methyltransferase (COMT) polymorphism is associated with anxiety symptoms in breast cancer patients [32]. Brain-derived neurotrophic factor (BDNF) Val66Met polymorphism was significantly correlated with anxiety in patients with advanced gastric cancer [33]. Polymorphisms in the 5-hydroxytryptamine transporter gene-linked polymorphism region (5-HTTLPR) were associated with anxiety and depression in breast cancer patients [34, 35]. This study showed that ERCC1 rs3212986, ERCC1 rs3212986-rs11615, ERCC2 rs13181-rs3916874-rs238416 were associated with anxiety or depression in LC patients. These factors may help screen out lung cancer patients with a higher risk of anxiety and depression to carry out a personalized intervention.

The limitation of this study is that there is no accurate information about the evaluation of chemotherapy effect and tissue samples of patients, so it is impossible to explore the relationship between SNPs and chemotherapy effect or protein expression level of lung cancer, which limits the exploration of the underlying mechanisms of the association between ERCC1, ERCC2 SNPs, haplotypes, and quality of life of LC patients. A further study with more focus on the mechanism behind this association is therefore suggested. Furthermore, for complex phenotypes, the influence of genetic factors may be the synergistic effect of multiple SNPs or even the influence of multiple gene pathways. Therefore, these nine SNPs loci may not fully reflect the impact of genetic factors on the quality of life of lung cancer patients.

## Conclusion

SNPs and haplotypes of ERCC1 and ERCC2 were associated with different domains of QoL, depression and anxiety in LC patients.

As few studies have explored the genetic factors affecting the quality of life, we first proposed the correlation between ERCC1, ERCC2 gene polymorphisms, and the quality of life of lung cancer patients, which provides a new perspective for the study of LC patients’ quality of life.

## Supplementary Information

**Additional file 1.**

## Data Availability

The datasets used and/or analyzed during the current study are available from the corresponding author on reasonable request.

## Abbreviations

EORTC: European organization for research and treatment of cancer
ERCC1: Excision repair cross-complementing 1
ERCC2: Excision repair cross-complementing 2
iMLDR: Improved multiplex ligation detection reaction
LC: Lung cancer
NER: Nucleotide excision repair
OR: Odds ratio
QLQ-C30: Core quality of life questionnaire
QLQ-LC13: Quality of Life Questionnaire-Lung Cancer 13
QoL: Quality of life
SNP: Single nucleotide polymorphism
tag SNPs: Tag single nucleotide polymorphism
