# Supplementary material for: Genetic polymorphisms and haplotypes of ERCC1 and ERCC2 associated with quality of life, depression, and anxiety status among patients with lung cancer
Source: BMC Cancer. 2021 Jul 21;21:842. doi: 10.1186/s12885-021-08570-5 (PMC8293557; doi:10.1186/s12885-021-08570-5)
Supplement: Supplementary file 1 — Additional file 1. [file 12885_2021_8570_MOESM1_ESM.docx]

| **SNP** | **chr** | **Chr. Position** | **Gene** | **Ref mRNA** | **SNP Property** | **Alleles** | **Functional Change** |
| --- | --- | --- | --- | --- | --- | --- | --- |
| rs11615 | 19 | 45923653 | ERCC1 | NM_001166049.1 | synon_exon4 | C/T | p.=(Asn118Asn) |
| rs762562 | 19 | 45912343 | ERCC1 | NM_001166049.1 | 3'-UTR | A/G |  |
| rs3212986 | 19 | 45912736 | ERCC1 | NM_001166049.1 | 3'-UTR | C/A |  |
| rs13181 | 19 | 45854919 | ERCC2 | NM_000400.3 | nonsynon_exon23 | T/G | p.Lys751Gln |
| rs171140 | 19 | 45865002 | ERCC2 | NM_000400.3 | intron11 | C/A |  |
| rs50871 | 19 | 45862515 | ERCC2 | NM_000400.3 | intron12 | C/A |  |
| rs50872 | 19 | 45862449 | ERCC2 | NM_000400.3 | intron12 | A/G |  |
| rs3916874 | 19 | 45856926 | ERCC2 | NM_000400.3 | intron17 | C/G |  |
| rs238416 | 19 | 45857049 | ERCC2 | NM_000400.3 | intron17 | T/C |  |

Supplementary Table 1 Specific information of SNPs
